# Quantitative analysis by next generation sequencing of hematopoietic stem and progenitor cells (LSK) and of splenic B cells transcriptomes from wild-type and *Usp3*-knockout mice

**DOI:** 10.1016/j.dib.2015.12.049

**Published:** 2016-01-08

**Authors:** Cesare Lancini, Gaetano Gargiulo, Paul C.M. van den Berk, Elisabetta Citterio

**Affiliations:** a Division of Molecular Genetics, The Netherlands Cancer Institute, Plesmanlaan 121, 1066 CX Amsterdam, The Netherlands; b Division of Biological Stress Response, The Netherlands Cancer Institute, Plesmanlaan 121, 1066 CX Amsterdam, The Netherlands

**Keywords:** BM, bone marrow, DDR, DNA damage response, DUBs, deubiquitinating enzymes, HSC, hematopoietic stem cell, LSK, Lin-Sca1+ cKit+ cells, RNA-seq, RNA sequencing, Ub, ubiquitin, USP3, Ub-specific protease 3, B cell, Deubiquitinating enzymes (DUBs), DNA damage response (DDR), Hematopoietic stem and progenitor cells (HSPC), Hematopoietic stem cells (HSC), LSK, RNA-seq, Transcriptional profiling, Ubiquitin, Ubiquitin-specific protease 3 (USP3), Histone H2A, DNA double-strand breaks (DSBs)

## Abstract

The data described here provide genome-wide expression profiles of murine primitive hematopoietic stem and progenitor cells (LSK) and of B cell populations, obtained by high throughput sequencing. Cells are derived from wild-type mice and from mice deficient for the ubiquitin-specific protease 3 (USP3; *Usp3Δ*/*Δ*). Modification of histone proteins by ubiquitin plays a crucial role in the cellular response to DNA damage (DDR) (Jackson and Durocher, 2013) [1]. USP3 is a histone H2A deubiquitinating enzyme (DUB) that regulates ubiquitin-dependent DDR in response to DNA double-strand breaks (Nicassio et al., 2007; Doil et al., 2008) [2], [3]. Deletion of USP3 in mice increases the incidence of spontaneous tumors and affects hematopoiesis [4]. In particular, *Usp3*-knockout mice show progressive loss of B and T cells and decreased functional potential of hematopoietic stem cells (HSCs) during aging. USP3-deficient cells, including HSCs, display enhanced histone ubiquitination, accumulate spontaneous DNA damage and are hypersensitive to ionizing radiation (Lancini et al., 2014) [4]. To address whether USP3 loss leads to deregulation of specific molecular pathways relevant to HSC homeostasis and/or B cell development, we have employed the RNA-sequencing technology and investigated transcriptional differences between wild-type and *Usp3*Δ/Δ LSK, naïve B cells or *in vitro* activated B cells. The data relate to the research article “Tight regulation of ubiquitin-mediated DNA damage response by USP3 preserves the functional integrity of hematopoietic stem cells” (Lancini et al., 2014) [4]. The RNA-sequencing and analysis data sets have been deposited in NCBI׳s Gene Expression Omnibus (Edgar et al., 2002) [5] and are accessible through GEO Series accession number GSE58495 (http://www.ncbi.nlm.nih.gov/geo/query/acc.cgi?acc=GSE58495). With this article, we present validation of the RNA-seq data set through quantitative real-time PCR and comparative analysis.

## Specifications Table

**Table:** 

Subject area	*Biology, Epigenetic*
More specific subject area	*Hematopoiesis, DNA damage, deubiquitinating enzymes (DUBs)*
Type of data	*Text file, graph, figure, table*
How data was acquired	*High-throughput RNA sequencing using the Illumina HiSeq 2000 (Mus musculus). qRT-PCR by SYBR Green assays.*
Data format	*Raw, processed and analyzed*
Experimental factors	*Cell isolation by FACS sorting, cell culture of LPS-stimulated B cells, preparation of RNA libraries by standard Illumina TruSeq RNA Sample Prep protocols.*
Experimental features	*Genome-wide expression profiling of mouse hematopoietic stem and progenitor cells (LSK cells), naïve splenic B cells and in vitro lipopolysaccharide (LPS)- stimulated B cells (activated B cell).* qRT–PCR on LSK cells by SYBR Green assays.
Data source location	*Amsterdam, The Netherlands*
Data accessibility	*The raw data discussed in this publication have been deposited in NCBI’s Gene Expression Omnibus*[5]*and are accessible through GEO Series accession no. GSE58 495. Validation of the RNA-seq data sets is available with this article.*

Direct link to deposited data: http://www.ncbi.nlm.nih.gov/geo/query/acc.cgi?acc=GSE58495

## Value of the data

• One of the largest dataset of gene expression profiles by RNA-seq of hematopoietic stem and progenitor cells (LSK) and B cells from mice deficient for a deubiquitinating enzyme available to date, and the first dataset for *Usp3* deletion.
• The data can be used to link transcriptional expression profiles to functional alterations in ubiquitin-regulated pathways and DNA damage response pathways
• The data can be compared to available transcriptional data for further insights into regulatory networks of hematopoiesis
• Data can be used in the development of further experiments aimed at addressing how the ubiquitin-dependent DNA damage response pathway impact on hematopoietic stem cell biology

## 1 Data

**Fig. 1:**
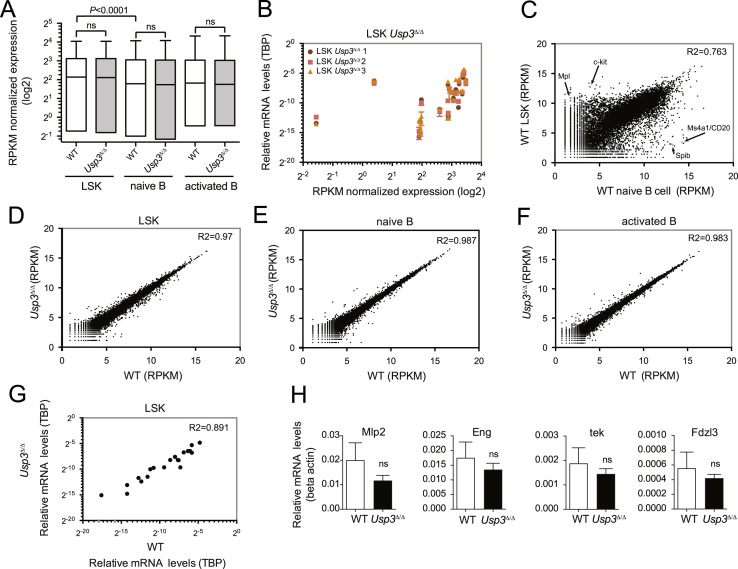
Analysis and validation of RNA-seq data from WT and *Usp3Δ*/*Δ* mice. (A) Expression levels of WT and *Usp3Δ*/*Δ* freshly isolated LSK and naïve B cells, and in vitro lipopolysaccharide (LPS) stimulated B cells (activated B cell). Statistical significant difference between the transcriptional profile of LSK versus naïve B cells is shown: ****P<0.0001. RPKM Reads Per Kilobase of transcript per Million mapped reads. (B) Expression of a set of 19 genes (Supplementary Table S1) was assessed by qRT-PCR in three independent LSK mRNA per each genotype. Results for the *Usp3Δ*/*Δ* genotype are shown. qRT-PCR and the RNA-seq normalized expression data for these genes in *Usp3Δ*/*Δ* samples had a good linear relationship, validating the RNAseq analysis. Pearson coefficient of LSK RNA-seq *Usp3Δ*/*Δ* compared with LSK qRT-PCR of: *Usp3Δ*/*Δ* 1, *r*=0.4853; *Usp3Δ*/*Δ* 2, *r*=0.4777; *Usp3Δ*/*Δ* 3, *r*=0.4964. *P<0.05. (C) Comparison of normalized gene expression data for WT LSK versus WT naïve B cells of one representative experiment. Pearson coefficient *r*=0.874. Distinct LSK-specific and B-cell specific expressed genes are recognizable. Among the LSK-specific genes, the Mpl receptor [6] and the Kit receptor [7] are indicated (gray dots). MS4A1/CD20 and Spi-B transcription factor are genes specifically expressed in B cells (gray dots). (D–F) Comparison of normalized gene expression data for *Usp3Δ*/*Δ* versus WT LSK (Pearson coefficient *r*=0.986, *R*^2^ coefficient=0.9738) (D), naïve B cell (Pearson coefficient *r*=0.974, *R*^2^=0.987) (E) and LPS activated B cell (Pearson coefficient *r*=0.991, *R*^2^ coefficient=0.983) (F). One representative experiment out of two is shown. (G) qRT-PCR analysis of a set of 19 genes in *Usp3Δ*/*Δ* and WT LSK (Supplementary Table S1). Pearson coefficient *r*=0.9443; *R*^2^ coefficient=0.891. Single qRT-PCR results for a subset of HSC-specific genes, Mpl2, Eng, Tek and Fdzl3 [8], is shown in (H). Data are from three independent LSK mRNA per genotype. Data are means±SEM. (A), (C–F) Two independent experiments were performed. For each experiment, pooled cells from 4 individual mice/genotype (8 weeks old) were analyzed by RNA-seq. Cells were: LSK, FACS-sorted LSKs from bone marrow; naïve B, FACS-sorted B cells from spleen (CD19+); activated B, B cells harvested and FACS sorted after 4 days stimulation with LPS in culture. (B), (G) qRT-PCR was performed on mRNA from FACS sorted LSK. *N*=three individual mice/genotype (8 weeks old).

mRNA profiles of murine populations of lineage negative cKit+ SCA1+ (LSK) hematopoietic progenitors, and naïve or activated B cell populations from 8 weeks-old wild-type (WT) and *Usp3*-deleted (*Usp3Δ/Δ*) mice were generated by deep sequencing using Illumina Hiseq2000. Here we present validation of the datasets by comparative analysis and qRT-PCR (Fig. 1). qRT-PCR validation of the RNA-seq on LSK cells was performed using SYBR Green assays.

## 2 Experimental design and materials and methods

### 2.1 Experimental design

The cells used as a source for RNA-seq were lineage negative, cKit+, SCa1+ (LSK) hematopoietic stem and progenitor cell population purified by fluorescence-activated cell sorting (FACS) from freshly isolated bone marrow, FACS-sorted naive B cells from spleens (CD19+) and activated B cells harvested and FACS sorted after 4 days stimulation with lipopolysaccharide (LPS) in culture. Two biological replicas were analyzed. For each experiment WT *n*=4, *Usp3*Δ*/*Δ** *n*=4 mice were used. FACS-sorted cells from individual animals were pooled and subjected to deep sequencing. We assigned about 8–16 million reads per sample uniquely to a gene of the mouse reference genome (mm9). We identified 23,429 genes in the LSKs, naive B cells and activate B cells of WT and *Usp3*Δ*/*Δ** mice using TopHat in combination with HTSeq-count. The raw data files that were used in the validation/analysis presented here and in the analysis and interpretation in [4] have been deposited in the NCBI’s Gene Expression Omnibus [5] database with the GEO Series accession no. GSE58 495 (Fig. 1).

### 2.2 Lin-Sca1+ cKit+ (LSK) hematopoietic progenitor isolation

Bone marrow cells were extracted from 8 weeks-old wild-type (WT) and Usp3 knockout (*Usp3Δ*/*Δ*) mice (MGI:5490048; B6.129P2(FVB)-Usp3<tm1.1Nki>) [4]. For cell sorting of bone marrow (BM) LSK sub-populations [9], [10], depletion of lineage+ cells was performed using Biotin MicroBeads (130-090-485, Macs, Miltenyi biotec) and magnetic columns (130-042-401, Macs, Miltenyi biotec). Cell were then directly stained with fluorochrome-conjugated antibodies against Sca1 (Pacific blue)(Biolegend), c-Kit (APC)(BD) and Strep (APC/CY7) (Souther Biotech). Cell sorting was performed with FACSAria (BD Biosciences). All animal experiments comply with local and international regulations and ethical guidelines and have been authorized by our local experimental animal committee at The Netherlands Cancer Institute (DEC-NKI).

### 2.3 B cell isolation

B cells were extracted from 8 weeks-old WT and *Usp3Δ*/*Δ* mice. Naive splenic B cells were obtained by CD43 depletion using biotinylated anti CD43 (Clone S7, BD Biosciences), and the IMag system (BD Biosciences), as described by the manufacturer. Naïve B cells were directly FACS sorted with fluorochrome-conjugated antibody specific for CD19 (APC), or cultured in vitro for four days in IMDM+8%FBS and 50 μg/ml *Escherichia coli* LPS (055:B5, Sigma) to obtain activated B cells, followed by sorting. Two independent experiments were performed. Cell sorting was performed by FACSAria (BD Biosciences).

### 2.4 RNA-seq gene expression analysis

For gene expression analysis, LSKs (FACS sorted from freshly isolated BM), naïve splenic B cells (freshly FACS sorted) or FACS sorted LSP-activated B cells were used. *N*=4 *Usp3Δ*/*Δ* and *N*=4 WT littermates (8 weeks-old). Cells from individual animals were pooled and total RNA was extracted. Samples were prepared using TruSeq protocols and standard Illumina sample preparation protocols and RNA-seq was performed on an Illumina Hiseq2000 machine at the NKI Genomics Core Facility. The sequence reads that passed quality filters were mapped to mm9 with TopHat version 2.0.3 and the gene expressions were calculated using HTSeq-count. The expression levels are normalized to 10 million reads per sample (GSE58495_diffexp_LSK.txt.gz file). Differential expression was performed using the R package DEGseq (GSE58495_norm_gene_exp_10mil.txt.gz). Upon differential expression analysis all values were added with 1. Genes that had no expression in both samples were removed.

### 2.5 Quantitative real time- (qRT-)PCR

Total RNA was extracted using Trizol reagent (Life technologies) and cDNA was prepared using Superscript II RT and oligod(T)n primers (Life technologies). qRT-PCR was performed on a StepOnePlus Real-Time PCR system (Applied Biosystems) using SYBR Green PCR mastermix (Applied Biosystems). The amount of target, normalized to an endogenous reference (TBP or beta actin) was calculated by: 2^−*ΔΔ*^ CT.

Primer sequences used in validation of RNA seq analysis (Supplementary Table S1) are available upon request.

### 2.6 Statistics

Statistical analysis was performed by Student *t* test or Pearson correlation analysis in Prism 6.

## Conflict of interest

The authors declare no competing financial interests.

## Footnotes

Appendix A Supplementary data associated with this article can be found in the online version at doi:10.1016/j.dib.2015.12.049.
