# Supplementary material for: Quantitative analysis by next generation sequencing of hematopoietic stem and progenitor cells (LSK) and of splenic B cells transcriptomes from wild-type and Usp3-knockout mice
Source: Data Brief. 2016 Jan 8;6:556–61. doi: 10.1016/j.dib.2015.12.049 (PMC4731422; doi:10.1016/j.dib.2015.12.049)
Supplement: Supplementary file 1 — Supplementary material [file mmc1.docx]

**Supplementary Table 1.**

List of genes validated by qRT-PCR in *Usp3Δ/Δ *and WT LSK.

| **Gene symbol** | **Gene name** |
| --- | --- |
| mpl | **myeloproliferative leukemia virus oncogene** |
| Eng | endoglin |
| tek | endothelial-specific receptor tyrosine kinase |
| Fdzl3 | frizzle |
| Fgf13 | fibroblast growth factor 13 |
| Atm | ataxia telangiectasia mutated homolog |
| p21 | cyclin-dependent kinase inhibitor 1A |
| Cd48 | CD48 antigen |
| Batf | basic leucine zipper transcription factor, ATF-like |
| Lyn | Yamaguchi sarcoma viral (v-yes-1) oncogene homolog |
| Ebf | early B cell factor 1 |
| Ighg1 | immunoglobulin heavy constant gamma 1 (G1m marker) |
| Cd37 | CD37 antigen |
| Trem | triggering receptor expressed on myeloid cells |
| Tlr13 | toll-like receptor 13 |
| Rnf128 | ring finger protein 128 |
| Ifng | Interferon gamma |
| Il11ra | interleukin 11 receptor, alpha chain 1 |
| Cxcl11 | chemokine (C-X-C motif) ligand 11 |

Primer sequences are available upon request.
